# The interleukin-4/interleukin-13 pathway in type 2 inflammation in chronic rhinosinusitis with nasal polyps

**DOI:** 10.3389/fimmu.2024.1356298

**Published:** 2024-04-16

**Authors:** Claus Bachert, Alexandra Hicks, Simon Gane, Anju T. Peters, Philippe Gevaert, Scott Nash, Julie E. Horowitz, Harry Sacks, Juby A. Jacob-Nara

**Affiliations:** ^1^ Department of Otorhinolaryngology – Head and Neck Surgery, University Hospital of Münster, Münster, Germany; ^2^ Sun Yat-sen University, International Airway Research Center, Guangzhou, China; ^3^ Immunology & Inflammation, Sanofi, Cambridge, MA, United States; ^4^ The Royal National Throat, Nose and Ear Hospital, London, United Kingdom; ^5^ Feinberg School of Medicine, Northwestern University, Chicago, IL, United States; ^6^ Upper Airways Research Laboratory, Faculty of Medicine, Ghent University, Ghent, Belgium; ^7^ Medical Affairs, Regeneron Pharmaceuticals Inc., Tarrytown, NY, United States; ^8^ Global Medical Affairs, Sanofi, Bridgewater, NJ, United States

**Keywords:** chronic rhinosinusitis with nasal polyps, CRSwNP, type 2 inflammation, cytokine, interleukin-4, interleukin-13, interleukin-4 receptor, biologic

## Abstract

Chronic rhinosinusitis with nasal polyps (CRSwNP) is predominantly a type 2 inflammatory disease associated with type 2 (T2) cell responses and epithelial barrier, mucociliary, and olfactory dysfunction. The inflammatory cytokines interleukin (IL)-4, IL-13, and IL-5 are key mediators driving and perpetuating type 2 inflammation. The inflammatory responses driven by these cytokines include the recruitment and activation of eosinophils, basophils, mast cells, goblet cells, M2 macrophages, and B cells. The activation of these immune cells results in a range of pathologic effects including immunoglobulin E production, an increase in the number of smooth muscle cells within the nasal mucosa and a reduction in their contractility, increased deposition of fibrinogen, mucus hyperproduction, and local edema. The cytokine-driven structural changes include nasal polyp formation and nasal epithelial tissue remodeling, which perpetuate barrier dysfunction. Type 2 inflammation may also alter the availability or function of olfactory sensory neurons contributing to loss of sense of smell. Targeting these key cytokine pathways has emerged as an effective approach for the treatment of type 2 inflammatory airway diseases, and a number of biologic agents are now available or in development for CRSwNP. In this review, we provide an overview of the inflammatory pathways involved in CRSwNP and describe how targeting key drivers of type 2 inflammation is an effective therapeutic option for patients.

## 1 Introduction

Chronic rhinosinusitis with nasal polyps (CRSwNP) is an inflammatory disease of the mucosa lining the nasal cavity and paranasal sinuses and is estimated to affect up to 4% of the adult population (1–3). The characteristic nasal polyps (NPs) are benign, edematous, inflammatory lesions that originate from the mucosa of the paranasal sinuses (mainly ethmoid) and can obstruct both the nasal airway and the olfactory cleft (4).

The main symptoms of CRSwNP are nasal congestion, rhinorrhea, and loss of smell (1). Smell loss has a significant negative impact on health-related quality of life (HRQoL) and is reported by patients to be one of the most important and troublesome symptoms of their disease (3, 5–8). Patients with CRSwNP experience a high symptom burden, which significantly impacts both their mental and their physical HRQoL (1, 9). Furthermore, patients often have coexisting inflammatory respiratory diseases, notably asthma and nonsteroidal anti-inflammatory drug-exacerbated respiratory disease (NSAID-ERD) (1). The standard of care for CRSwNP includes intranasal corticosteroids and saline irrigation (3, 10). Sinonasal surgery or oral steroids are needed for patients with uncontrolled and more severe disease. However, recurrence is common, with up to 40% of patients needing further surgery within 18 months (2, 11). Oral steroids may provide short-term relief, although repeated courses are associated with adverse effects including but not limited to weight gain, high blood pressure, and an increased risk of infections.

Although not fully elucidated, it is thought that environmental factors (airborne fungi, superantigenic exotoxins produced by *Staphylococcus aureus*, and pro-inflammatory biofilms) and individual susceptibility (epithelial barrier dysfunction, innate immunity, and genetics) contribute to the pathogenesis of CRSwNP, with nasal microbiota dysbiosis influencing the inflammatory mechanism determining type 1 or type 2 predominance (12–18). Recent studies have shed further light on the molecular diversity of CRSwNP, with evidence of distinct disease endotypes (19–22). An increased understanding of the molecular mechanisms involved in the pathogenesis of CRSwNP and of the predominant role of type 2 inflammation has led to the development of biologic therapies to block specific type 2 inflammatory pathways in CRSwNP. This review provides an overview of the inflammatory pathways involved in CRSwNP and describes how targeting key drivers of type 2 inflammation is an effective therapeutic option for patients with CRSwNP whose disease is uncontrolled or poorly controlled with standard therapeutic approaches.

## 2 Immune defense in the airway mucosa

The cells and tissues of the airway mucosal lining act as the first line of defense against airborne pathogens, irritants, and other harmful particles. Penetration of the mucosal barrier that results in infection or damage can trigger an acute inflammatory response involving immune T cell activation and the release of cytokines and other locally acting messengers that induce the local accumulation of immune cells (23–25).

Inflammatory responses can be divided into three types. Type 1 inflammation is primarily involved in host defense against viral, bacterial, and fungal pathogens and is mediated by T helper (Th)1 and Th17 T cells and by type 1 innate lymphoid cells (ILCs). The key cytokines involved in type 1 inflammatory responses include interferon gamma and tissue necrosis factor alpha. Type 2 inflammation is involved in defense against parasitic infections and is also a primary driver of allergic disease. Type 2 inflammation is mediated by various immune cells including Th2 T cells, type 2 ILCs, eosinophils, mast cells, basophils, and immunoglobulin E (IgE)-secreting B cells. The key cytokines involved in type 2 inflammatory responses are interleukin (IL)-4, IL-5, and IL-13. IL-4 and IL-13 are involved in polyclonal IgE formation. IL-4/IL-13 signaling drives class switching of B cells to IgE production (26). Type 3 inflammation is primarily involved in host defense against bacterial and fungal pathogens and is mediated by Th17 cells and type 3 ILCs. The key cytokines involved in type 3 inflammatory responses are IL-17 and IL-22.

## 3 Pathophysiology of CRSwNP

The chronic inflammatory processes associated with CRSwNP result in cellular changes and structural remodeling of the nasal mucosa (27). These changes consist of morphologic and functional changes to the nasal epithelial cells, including proliferation of basal cells, hyperplasia of mucus-secreting goblet cells, and an epithelial–mesenchymal transition with loss of the differentiation of the ciliated cells at the mucosal surface (27). These changes compromise the barrier function and the regenerative capacity of the nasal epithelium and reduce mucociliary clearance. Degradation of the extracellular matrix also contributes to reduction of the structural integrity of the nasal mucosa, a process that is accompanied by fibrin deposition and tissue edema. Abnormal and excessive fibrin deposition occurs within both the nasal mucosa and the NPs and contributes to the retention of plasma proteins, localized edema, and increased viscosity and adherence of nasal mucus (28). Changes to the neuronal supply also occur, with a decrease in the quantity of olfactory neurons (29) and a switch in olfactory stem cells from neuroregeneration to immune defense (30).

In the West, most patients with CRSwNP display type 2 inflammation, characterized by elevated levels of type 2 cytokines (21). Cluster and gene expression analyses have been conducted to further our understanding of the different endotypes in chronic rhinosinusitis (19–21). One study showed that clusters with a type 2 inflammatory signature were associated with a mixture of phenotypes in which there was 47–64% and 20–37% prevalence of CRSwNP and comorbid asthma, respectively (19). Another cluster analysis identified two clusters that carried a predominantly type 2 signature (which included high levels of IL-5, IL-4, and IL-13), with all patients having CRSwNP, a high frequency of comorbid asthma (>80%), high disease burden including smell impairment, and a high incidence of surgery (20). In a study that used mRNA and protein to identify endotypes in chronic rhinosinusitis, a type 2 signature was predominant in both phenotypes of chronic rhinosinusitis and was associated with loss of smell and asthma comorbidity (21). Type 2 pathophysiology is shared by several other atopic diseases, including asthma, NSAID-ERD, atopic dermatitis (AD), and allergic rhinitis, and these conditions often co-occur. The prevalence of asthma among patients with CRSwNP may be as high as 65%, and the prevalence of NSAID-ERD may be as high as 26% (1, 31). The prevalence of AD among patients with CRSwNP is much lower (9% (31)), although patients with CRSwNP do appear to be at an increased risk of developing AD (32). Almost three-quarters of CRSwNP patients have comorbid allergic rhinitis (31), and up to 86% of patients with CRSwNP are estimated to be sensitized to at least one aeroallergen, with the possibility of symptoms of CRSwNP worsening during allergen season (33).

In East Asia, patients with CRSwNP often display type 1 or a mixed type 1/2 inflammatory pattern, suggesting genetic factors may also play a role (34–36). However, in recent years, there appears to have been a shift toward increasing involvement of type 2 inflammation (19, 22, 37).

### 3.1 Mechanisms of type 2 inflammation in CRSwNP

**Figure 1:**
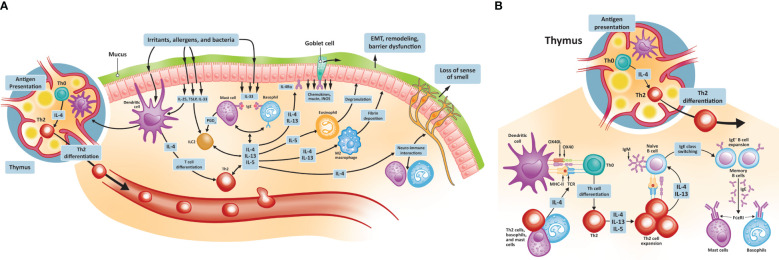
Type 2 inflammatory pathways in CRSwNP. **(A)** Type 2 inflammatory pathways at the nasal epithelium and thymus; **(B)** Th2 cell differentiation and activation. CRSwNP, chronic rhinosinusitis with nasal polyps; EMT, epithelial-to-mesenchymal transition; FCεRI, immunoglobulin E receptor; IgE, immunoglobulin E; IL, interleukin; iNOS, inducible nitric oxide synthase; OX40L, OX40 ligand; MHC, major histocompatibility complex; TCR, T cell receptor; Th, T helper; TSLP, thymic stromal lymphopoietin.

Type 2 inflammation in CRSwNP is characterized by the hyperproduction of IgE, eosinophilic inflammation, and mucus hyperproduction (38). The signaling pathways that initiate these changes are stimulated following injury of the damaged epithelium by pathogens, proteases, and irritants (**Figure 1**). These insults lead first to the production of the cytokines IL-25, IL-33, and thymic stromal lymphopoietin (TSLP) by epithelial cells, which in turn promote the development of mature Th2 cells. TSLP is the most highly induced of these cytokines and activates type 2 ILCs and mast cells. TSLP also promotes the release of additional type 2 cytokines, notably IL-4, IL-5, and IL-13, thereby amplifying the type 2 inflammatory response. IL-33 mediates eosinophilic infiltration of the nasal mucosa and can also induce IL-4 and IL-5 production from eosinophilic NPs (39, 40). As Th2 cells mature, they too begin to secrete a range of cytokines, including IL-4, IL-5, and IL-13. IL-4 and IL-13 drive the key features of type 2 inflammation, including innate immune cell recruitment to sites of inflammation (41, 42). These immune cells include mast cells, eosinophils, basophils, and M2 macrophages. A key type 2 inflammatory response is B cell differentiation into IgE-producing plasma cells. IgE binding to mast cells induces further cytokine production, especially IL-5, which acts to stimulate eosinophil production in the bone marrow and, together with IL-4 and IL-13, promotes trafficking of eosinophils to sites of tissue inflammation, thereby driving eosinophilic inflammation. IL-4 and IL-13 promote eosinophil trafficking by inducing the production of eosinophil-promoting factors [including IL-5 and eotaxin-1 (43)]. IL-13 also plays a specific role through the stimulation of processes resulting in local tissue remodeling (44–46), including mucus hyperproduction by goblet cells. While under nonpathogenic conditions, these inflammatory responses would be downregulated, patients with CRSwNP continue to have elevated levels of IL-4, IL-13, and IL-5 in both eosinophilic and non-eosinophilic NP tissue, compared with healthy controls (47, 48). Local colonization by *S. aureus* may play a role in CRSwNP pathogenesis, with enterotoxins secreted by *S. aureus* acting as superantigens that amplify immune responses and shift inflammation toward the type 2 signature (49, 50). Levels of IgE antibodies to staphylococcal enterotoxin superantigens in NP tissue may be associated with disease severity (51).

Type 2 inflammation contributes to NP formation. IL-4 and IL-13 appear to promote NP formation by increasing the expression of periostin, an extracellular matrix protein involved in fibrotic airway tissue remodeling (52, 53). Periostin is secreted by lung fibroblasts of individuals with asthma and acts as an adhesion molecule, binding other extracellular matrix proteins and resulting in subepithelial fibrosis (53). A similar process of increased extracellular protein adhesion, fibrosis, and consequent edema may be involved in NP formation. Periostin is secreted by nasal epithelial cells and, in addition to promoting tissue remodeling, can stimulate the secretion of TSLP, thereby contributing to the continuation of the exaggerated type 2 inflammatory process (54). Among patients with CRSwNP, the expression of periostin is higher in the NPs of those with coexistent asthma than those without and is positively associated with levels of TSLP (54).

### 3.2 Chronic inflammation and loss of smell in CRSwNP

Smell loss is a symptom associated with considerable decrement to patients’ HRQoL and imposes an increased risk of depression and anxiety (29, 55, 56). Effective treatments are limited, even in the absence of the additional symptoms associated with CRSwNP (56).

Mechanical obstruction of the airflow to the olfactory cleft due to edema, mucus discharge, and/or the physical presence of NPs may contribute to a loss of smell in patients with CRSwNP (29). However, mechanical obstruction is not thought to be the principal cause of loss of smell, and the physical removal of NPs to improve the airflow to the olfactory cleft does not always restore the sense of smell (57, 58). Indeed, patients with chronic rhinosinusitis without NPs may also experience smell loss, indicating the involvement of additional mechanistic factors.

Type 2 inflammation is implicated in the loss of smell through a variety of different mechanisms (29). The histologic changes that result from type 2 inflammation may decrease the number and availability of olfactory neurons and impact their function (59, 60). Recent data support IL-4 playing a direct role in impairing the function of olfactory neurons (61). In mice, IL-4 and IL-13 each increased the calcium uptake in primary olfactory sensory neurons *in vitro*, while the direct intranasal application of IL-4, but not IL-13, resulted in a rapid loss of smell *in vivo* (61). These findings suggest nonredundant effects of these cytokines on olfactory function. IL-4-induced loss of smell in this mouse model was attenuated in IL-4 receptor (R)α knockout mice and by treatment with IL-4Rα antibody in wild-type mice. Type 2 inflammation may also impact the formation of new olfactory sensory neurons. Under normal, nonpathogenic conditions, the nasal mucosa is capable of effective regeneration of cells, including olfactory neurons, from horizontal basal cells (30). However, under conditions of chronic type 2 inflammation, the regenerative capacity of the nasal mucosa is lost, as the differentiation of horizontal basal cells is inhibited (30). This direct impact on the formation of new olfactory sensory neurons was demonstrated in a mouse model of Th2‐mediated allergic chronic rhinosinusitis in which a reduction in the number of immature olfactory neurons was observed, while the number of mature olfactory neurons was unchanged (62). Another transgenic mouse model study found that induced expression of IL-13 within the olfactory epithelium resulted in time-dependent loss of neurons from the olfactory epithelium, as well as increased horizontal basal cell proliferation, mucus production, and expression of eotaxin (63). Thus, it appears that chronic type 2 inflammation, typified by elevated IL-4, IL-5, and IL-13, results in impaired olfactory neurogenesis. In addition to type 2 inflammation, there is evidence that type 1 inflammatory cytokines may also contribute to olfactory loss in CRSwNP, indicating a potential role of mixed inflammation (64, 65).

The long-term loss of smell associated with type 2 inflammation in CRSwNP is an area of considerable unmet need, and the potential for recovery of olfactory function is an ongoing area of research (55, 56). As insights emerge about the role of the various signaling pathways of type 2 inflammation, novel targets for therapeutic intervention are becoming apparent, one of which is the IL-4/IL-13 signaling pathway.

### 3.3 Cytokine signaling pathways in CRSwNP

The earliest components of the cytokine cascade that drives type 2 inflammation in CRSwNP are IL-25, IL-33, and TSLP. These cytokines are released by injured epithelial cells and bind to the respective receptors, namely IL-25R, IL-33R, and TSLPR on the surface of type 2 ILCs (66). Binding to the nascent receptors initiates intracellular signaling, resulting in the production and release of the type 2 cytokines IL-4, IL-5, and IL-13.

The biologic effects of IL-4 are mediated via two types of cell surface receptor. The type I receptor complex binds only IL-4 and consists of an IL-4Rα subunit associated with an accessory common gamma chain. The gamma chain is expressed mainly in hematopoietic cells, with little or no expression on nonhematopoietic cells. The type II receptor complex is a dimeric unit that consists of an IL-4Rα subunit and an IL-13Rα1 subunit and binds both IL-4 and IL-13. IL-13Rα1 is widely expressed in nonhematopoietic cells, although only low levels are expressed by lymphocytes, which predominantly express the gamma chain (67). While IL-13 is able to bind to and initiate intracellular signaling via the type II receptor complex, IL-13 is also able to bind another receptor, IL-13Rα2. IL-13Rα2 is a monomeric receptor that may also act as a scavenger or decoy receptor, preventing the dimerization of the type II receptor complex and acting as a negative regulator of IL-13-mediated signaling (67). The overlapping functions of IL-4 and IL-13 in driving type 2 inflammatory responses are in part due to the shared receptor component, with differences in components of the downstream signaling cascade thought to be responsible for the distinct activities driven by these two cytokines (66, 67). IL-5 binds to IL-5Rα, which forms a complex with a beta chain (66).

Receptor binding of IL-4, IL-13, or IL-5 triggers intracellular signaling cascades mediated by members of the Janus kinase (JAK)-signal transducer and activator of transcription (STAT) family of proteins (42, 66). JAK activation results in phosphorylation of specific amino acid residues that then act as docking sites for STATs and other signaling molecules. STATs are soluble transcription factors that, once activated at the receptor, translocate to the nucleus where they induce gene expression. Different STATs drive the expression of different sets of genes; for example, STAT6 increases the expression of the *eotaxin-1* gene, which encodes eotaxin, a powerful eosinophil chemotactic protein (68). IL-4 binding to the type I receptor leads to JAK1/JAK3 activation and subsequent activation of STAT6. Binding to the type II receptor by IL-4 (or IL-13) results in activation of JAK1/tyrosine kinase 2 and STAT6 (67, 69). On binding to its receptor, IL-5 activates the JAK1/JAK2 pathway, with subsequent activation of STAT1, STAT5, and STAT3 (66, 70, 71).

## 4 Therapeutic targeting of the key drivers of type 2 inflammation in CRSwNP

Several therapies targeting type 2 inflammation are approved or are under investigation for CRSwNP (**Table 1**). Targeting both IL-4- and IL-13-mediated signaling simultaneously has proved successful and has led to the approval of the first IL-4-/IL-13-targeted biologic agent for the treatment of CRSwNP: dupilumab. Dupilumab is a fully human monoclonal antibody that blocks IL-4Rα, the shared receptor component for IL-4 and IL-13, and therefore prevents signaling by both IL-4 and IL-13 (72, 73). Dupilumab is approved for the treatment of uncontrolled CRSwNP in adults and for other allergic diseases, including certain types of moderate-to-severe asthma, moderate-to-severe AD, eosinophilic esophagitis, and prurigo nodularis (74). Biomarker analysis has revealed that dupilumab treatment in patients with CRSwNP reduced concentrations of type 2 inflammatory biomarkers including eotaxin-3, periostin, eosinophil cationic protein (ECP), and IL-5 in nasal secretions, eotaxin-3, thymus and activation-regulated chemokine, periostin, and total IgE in blood, and leukotriene E4 in urine (75, 76). Dupilumab has also been found to downregulate local IgE production and eosinophil homing (66), and to decrease inflammatory eicosanoid levels and increase levels of anti-inflammatory prostaglandin E_2_ (77). Dupilumab treatment has been associated with rapid improvement in patient-reported sense of smell (78–80), which suggests the involvement of mechanisms other than improvements in airflow conduction and is consistent with preclinical findings showing direct roles of IL-4 and IL-13 on olfactory function (61, 63).

**Table 1:** Targeted biologic agents for CRSwNP.

Target	Agent	Type of monoclonal	Approval status in CRSwNP^a^
IL-4Rα (IL-4 and IL-13 signaling)	Dupilumab	IgG4/κ human	Approved (US, EU, and other territories)
	Rademikibart (CBP-201)	IgG4/κ human	Investigational (pre-Phase 3)
IL-5Rα	Benralizumab	IgG1/κ humanized	Investigational (Phase 3)
IL-5	Mepolizumab	IgG1/κ humanized	Approved (US, EU, and other territories)
	Depemokimab	IgG1/κ humanized	Investigational (Phase 3)
IgE	Omalizumab	IgG1/κ humanized	Approved (US, EU, and other territories)
IL-33	Etokimab	IgG1/κ humanized	Investigational (pre-Phase 3)
TSLP	Tezepelumab	IgG2/λ human	Investigational (Phase 3)

a January 2024.

IgG, immunoglobulin G; IgE, immunoglobulin E; IL-4Rα, interleukin-4 receptor alpha; IL-5Rα, interleukin-5 receptor alpha; IL-13, interleukin-13; TSLP, thymic stromal lymphopoietin.

Mepolizumab is an IL-5 antagonist monoclonal antibody that was approved first for severe asthma with eosinophilic inflammation. It recently received approval as an add-on maintenance treatment of CRSwNP in adults with inadequate response to intranasal corticosteroids (81). Mepolizumab induced sustained, significant reductions in blood eosinophil counts and reduced ECP and IL-5 receptor alpha levels in serum and IL-5 receptor alpha, IL-6, IL-1 beta, myeloperoxidase, and periostin levels in nasal secretions in patients with CRSwNP (82–84). Benralizumab is a humanized IL-5Rα-directed cytolytic monoclonal antibody that is approved for severe eosinophilic asthma (85) with one Phase 3 study in CRSwNP completed and another ongoing (86, 87). Benralizumab prevents binding of IL-5 to the IL-5Rα subunit and the subsequent heterodimerization with the beta chain subunit required to initiate IL-5-mediated signaling (88). Biomarker findings with benralizumab in CRSwNP are limited but in patients with asthma, benralizumab reduced blood eosinophil counts and reduced serum eosinophil-derived neurotoxin and ECP, and increased serum IL-5, eotaxin-1, and eotaxin-2 (89). Also under phase 3 evaluation is depemokimab, a humanized monoclonal antibody that blocks IL-5 from binding to the IL-5R complex (NCT05274750).

Omalizumab, a monoclonal antibody that targets IgE, is approved by the United States Food and Drug Administration for the treatment of CRSwNP based on the results of the phase 3 POLYP-1 and POLYP-2 studies (90). This agent acts downstream of the cytokine-mediated signaling that drives aberrant type 2 inflammation, acting instead on the effector molecule IgE, preventing the activation of immune cells including basophils and mast cells (91, 92). In patients with CRSwNP and asthma, omalizumab reduced eosinophil counts in blood and nasal smears (93).

**Figure 2:**
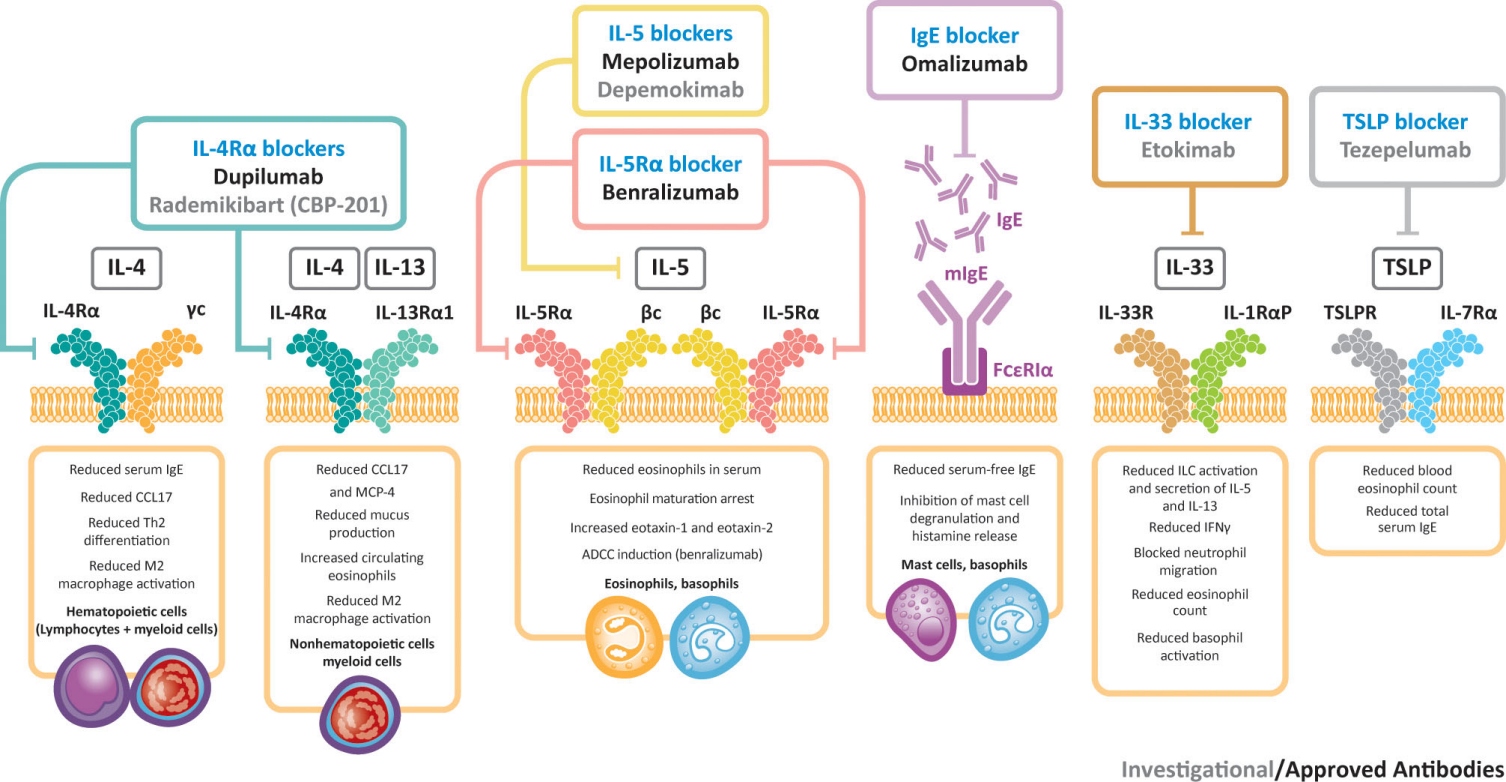
Biologics targeting type 2 inflammation in CRSwNP. βc, beta chain; γc, gamma chain; ADCC, antibody-dependent cellular cytotoxicity; CCL17, C–C motif chemokine ligand 17; IFNγ, interferon gamma; IgE, immunoglobulin E; IL, interleukin; ILC, innate lymphoid cell; MCP-4, monocyte chemoattractant protein-4; R, receptor; Th, T helper; TSLP, thymic stromal lymphopoietin.

Other biologic agents under evaluation for CRSwNP include etokimab (an IL-33-targeted agent) and tezepelumab (an agent that targets TSLP) (91). A phase 2 study of etokimab (NCT03614923) was completed in 2020, although future development plans for this agent have yet to be announced. A phase 3 study is underway for tezepelumab (NCT04851964) and data are expected in 2024. **Figure 2** presents a summary of biologics targeting type 2 inflammation in CRSwNP. An investigational IL-4Rα inhibitor, rademikibart (CBP-201), began investigation in a clinical trial in patients with CRSwNP in 2021, but the trial was terminated owing to enrollment challenges (94).

To date, no head-to-head clinical studies have been reported in CRSwNP directly comparing the efficacy of biologics targeted at different components of the type 2 inflammatory cascade. However, a recent network meta-analysis of the available evidence on biologics including dupilumab, omalizumab, mepolizumab, and benralizumab found clinically important differences in the effects among these agents: dupilumab ranked among the most beneficial for all outcomes studied (HRQoL, sinusitis symptoms, sense of smell, rescue oral corticosteroids, rescue nasal surgery, nasal polyp score, computed tomography score, and adverse events); with omalizumab among the most beneficial for sinusitis symptoms and HRQoL and intermediately beneficial for sense of smell, rescue surgery, and nasal polyp score; mepolizumab among the most beneficial for sinusitis symptoms and intermediately beneficial for HRQoL, sense of smell, rescue oral corticosteroids, rescue surgery, and nasal polyp score; and benralizumab intermediately beneficial for HRQoL, sense of smell, and rescue oral corticosteroids (95). Limitations of this analysis included small sample sizes and low event rates of rescue sinonasal surgery, rescue oral corticosteroids, and adverse events, which precluded precise effect estimates and high certainty.

## 5 Conclusion

CRSwNP is predominantly a type 2 inflammatory disease. Biologic agents targeting the signaling pathways underlying type 2 inflammation have demonstrated clinical efficacy in patients with moderate-to-severe CRSwNP, reducing symptoms and improving HRQoL.

## Author contributions

CB: Writing – original draft, Writing – review & editing. AH: Writing – original draft, Writing – review & editing. SG: Writing – original draft, Writing – review & editing. AP: Writing – original draft, Writing – review & editing. PG: Writing – original draft, Writing – review & editing. SN: Writing – original draft, Writing – review & editing. JH: Writing – original draft, Writing – review & editing. HS: Writing – original draft, Writing – review & editing. JJ-N: Writing – original draft, Writing – review & editing.
